# The Role of Sonic Hedgehog in Human Holoprosencephaly and Short-Rib Polydactyly Syndromes

**DOI:** 10.3390/ijms22189854

**Published:** 2021-09-12

**Authors:** Christine K. C. Loo, Michael A. Pearen, Grant A. Ramm

**Affiliations:** 1 South Eastern Area Laboratory Services, Department of Anatomical Pathology, NSW Health Pathology, Prince of Wales Hospital, Sydney, NSW 2031, Australia; 2 Hepatic Fibrosis Group, Department of Cell and Molecular Biology, QIMR Berghofer Medical Research Institute, Brisbane, QLD 4006, Australia; Michael.pearen@qimrberghofer.edu.au (M.A.P.); Grant.Ramm@qimrberghofer.edu.au (G.A.R.); 3 Faculty of Medicine, The University of Queensland, Brisbane, QLD 4006, Australia

**Keywords:** Sonic Hedgehog, human holoprosencephaly, short-rib polydactyly syndromes

## Abstract

The Hedgehog (HH) signalling pathway is one of the major pathways controlling cell differentiation and proliferation during human development. This pathway is complex, with HH function influenced by inhibitors, promotors, interactions with other signalling pathways, and non-genetic and cellular factors. Many aspects of this pathway are not yet clarified. The main features of Sonic Hedgehog (SHH) signalling are discussed in relation to its function in human development. The possible role of SHH will be considered using examples of holoprosencephaly and short-rib polydactyly (SRP) syndromes. In these syndromes, there is wide variability in phenotype even with the same genetic mutation, so that other factors must influence the outcome. *SHH* mutations were the first identified genetic causes of holoprosencephaly, but many other genes and environmental factors can cause malformations in the holoprosencephaly spectrum. Many patients with SRP have genetic defects affecting primary cilia, structures found on most mammalian cells which are thought to be necessary for canonical HH signal transduction. Although SHH signalling is affected in both these genetic conditions, there is little overlap in phenotype. Possible explanations will be canvassed, using data from published human and animal studies. Implications for the understanding of SHH signalling in humans will be discussed.

## 1. Sonic Hedgehog Signalling

The Hedgehog (HH) signalling pathway is one of the major pathways controlling cell patterning, differentiation and proliferation during human development. In adult life, HH signalling is involved in maintenance of tissue homeostasis, regeneration and neoplasia. Signalling is normally initiated by HH proteins, which act as activating ligands. There are three HH orthologs in vertebrates: SHH (Sonic Hedgehog), IHH (Indian hedgehog) and DHH (Desert hedgehog) [1,2]. The HH signalling pathway is complex and has a canonical pathway which requires functional primary cilia in vertebrates and non-canonical pathways that can occur both within and outside the cilia and involve modifiers and co-receptors. This review focuses on SHH and SHH signalling; however, downstream of the HH ligand, the molecular mechanisms of HH signalling are largely conserved; therefore, downstream aspects of SHH signalling also apply to HH signalling in general [2]. The function of Sonic Hedgehog (SHH) signalling in human patients will be explored in the examples of holoprosencephaly caused by genetic mutations in *SHH* and short rib polydactyly (SRP), caused by abnormalities in primary cilia.

SHH functions have been extensively investigated over more than four decades, initially in Drosophila [3]. However, the SHH pathway shows similarities between humans and many experimental models. It has been reported that there is 92.4% homology of SHH in mice and humans [4], and the main components of the canonical HH signalling pathway are evolutionarily conserved. Divergence between species is noted for some components of the SHH pathway, for example: the protein kinase FUSED and COS2 are not conserved, suggesting that HH signal transduction might differ in vertebrates and invertebrates. Furthermore, only a single Hedgehog gene (*Hh*) is known in Drosophila [1,2]. SHH polypeptides are produced in different cell types at different stages of development [1,5] and are involved in the development of almost all organs, including patterning of the brain and spinal cord, axial skeleton and limbs.

**Figure 1:**
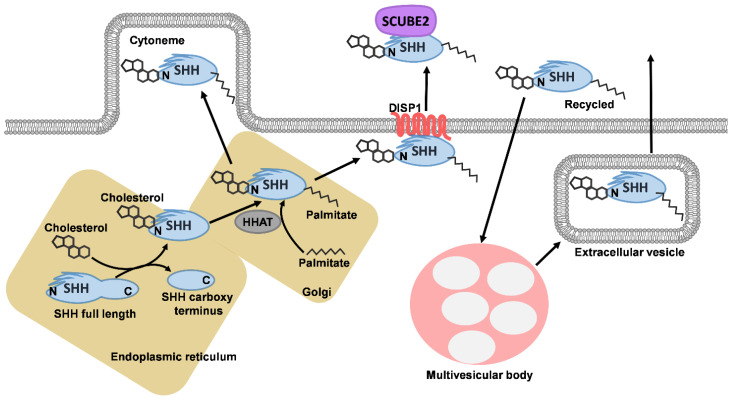
SHH production and secretion. SHH polypeptides are transported to the endoplasmic reticulum and golgi, where they are cleaved into the amino-terminal domain (which has signalling function) and carboxyl-terminal domain (autoprocessing regulation). The amino-terminal part is modified with palmitate (mediated by HHAT) and cholesterol (mediated by carboxyl end of the initial polypeptide). The active part of SHH may be transported to a cytoneme or bound to DISP1. SHH that has already been secreted can be recycled into the cell, transported to microvesicular bodies where they can be packaged into extracellular vesicles for secretion (modified after [10]).

SHH is secreted in sites with organising activity, for example, in the notochord and prechordal mesoderm, spreading to the overlying neural tube and patterns its dorsoventral axis. In the developing limb, SHH morphogen is produced by cells in the zone of polarising activity (ZPA) in the limb bud and patterns the anteroposterior axis of the limb and specifies digit number and identity [6]. *Shh*^−/−^ mouse models show abnormalities of the floorplate, neural tube and notochord in early embryos, with later defects observed in the distal limb structures, cyclopia, abnormalities of ventral cell types of the neural tube, absent spinal column and most of the ribs [7]. These polypeptides are transported to receiving cells by mechanisms that are currently not fully elucidated. There is evidence that morphogens such as SHH and Wnt are transported in extracellular vesicles [8] or possibly by other means, such as via lipoprotein particles, cytonemes in cells [6,9]. In the secreting cell (Figure 1), SHH polypeptides are transported to the endoplasmic reticulum and golgi, where they are cleaved into an amino-terminal domain (which has signalling function) and a carboxyl-terminal domain (autoprocessing regulation). The amino-terminal part is modified with palmitate and cholesterol. The carboxyl ends of the initial polypeptides act as cholesterol transferases and palmitoylation is mediated by HHAT (HH acyltransferase) in the endoplasmic reticulum [10]. SHH is bound to the cell membrane by modification with palmitate at the N-terminus and cholesterol at the C-terminus, which requires solubilising factors for release from producing cells, first binding with the membrane protein Dispatched 1 (DISP1) and then SCUBE2 (Signal peptide, CUB domain and EGF domain containing protein 2). DISP is necessary for long-range signalling of cholesterol modified SHH ligands although not for juxtacrine signalling [11]. 

## 2. Primary Cilia and Sonic Hedgehog Signalling

**Figure 2:**
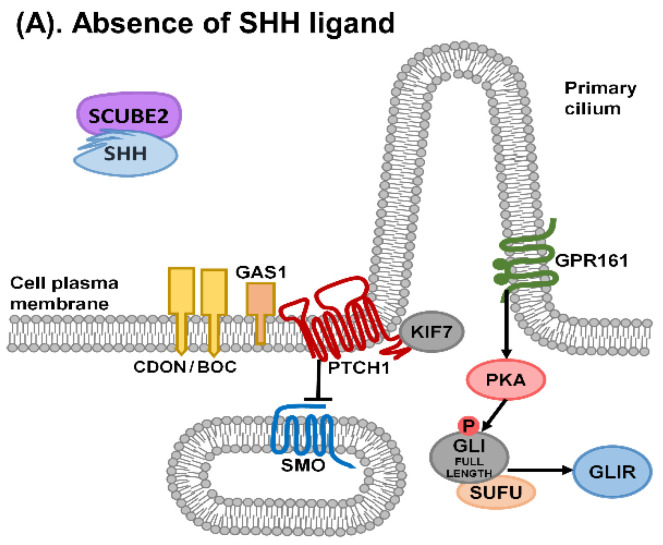

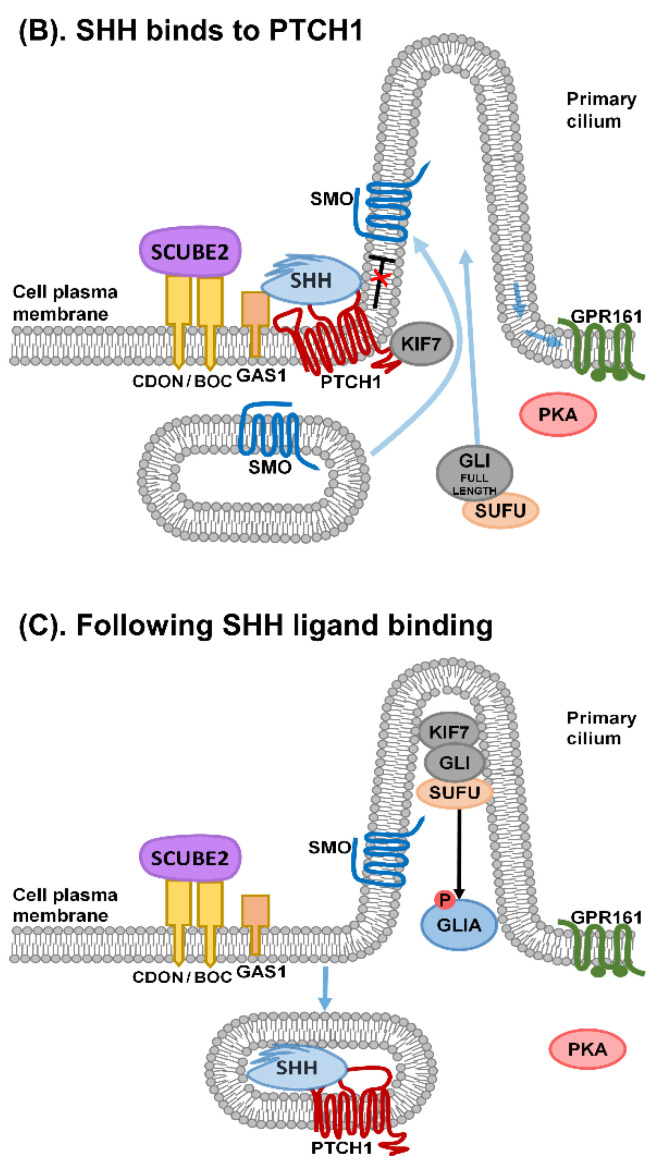
Canonical SHH signalling in primary cilia. (**A**). In the absence of SHH, PTCH1 at the base of the cilium blocks SMO from entry into the cilium. GPR161 activates PKA (protein kinase A), which in turn phosphorylates full-length GLI transcription factors. As a result, full-length GLI becomes cleaved by the proteasome at the base of the cilium to form GLIR (repressor form of GLI). (**B**). CDON/BOC and GAS1 are required for SHH signalling and the proposed role for these co receptors is outlined in [6]. On arrival at the target cell, the SCUBE2–SHH complex is bound by co receptors CDON/BOC, SHH is passed to GAS1 then to its receptor PTCH1. Binding of SHH to PTCH1 causes activation of SMO, resulting in the removal of GPR161 and reduction of PKA activity. Full length GLI is no longer cleaved to form GLIR. (**C**). When SHH binds to its receptor PTCH1, PTCH1 is endocytosed, releasing the inhibition on SMO. SMO accumulates in the cilium. A complex of SUFU, KIF7 and GLI is transported to the top of the cilium where the full length GLI is phosphorylated and converted to GLIA (activator form of GLI) (modified after [12,16,17]).

The canonical SHH signalling pathway is dependent on intact ciliary function in vertebrates, and major aspects of this pathway have recently been reviewed [12]. The canonical SHH signalling pathway is mediated via GLI transcription factors (Figure 2). Vertebrates have 2 Patched receptors (PTCH1 and PTCH2) for SHH ligands, but PTCH1 is the main receptor for SHH in vivo [13]. PTCH1 is normally located at the base of primary cilia on the target cell in the absence of the SHH ligand. In the absence of HH ligands, PTCH1 blocks Smoothened (SMO) from entering into the cilium. SCUBE2 forms a complex with lipidated SHH, but signalling by SCUBE-SHH requires co-receptors Cell adhesion molecule-related/downregulated by oncogenes/brother of CDON (CDON/BOC) and (Growth arrest specific 1) GAS1 for HH signalling. These co-receptors bind SHH and in their absence, SHH signalling is abolished. In addition, GAS1 and BOC each form distinct SHH receptor complexes with SHH [14]. CDON/BOC and GAS1 remove SHH from SCUBE, then transfer SHH to PTCH1 to initiate signalling. CDON/BOC and GAS1 have different actions and efficacy. GAS1 has a key role, and a more severe phenotype is seen in mutants with GAS1 loss compared with the loss of CDON/BOC in the neural tube and limb bud. In the limb bud, CDON/BOC are dispensable, but in the context of GAS1 loss, BOC can compensate weakly. In the neural tube, the loss of GAS1 resembles the loss of CDON/BOC (reviewed in [6]). Studies in mice show that *Gas1*, *Cdon* and *Boc* expression vary in time and spatial distribution and may regulate HH expression [15]. These co-receptors may regulate HH function in a time- and tissue-specific manner. However, it is uncertain if these co-receptors also localise to cilia [5]. HHIP (Hedgehog interacting protein), GAS1, CDON and BOC bind to SHH but are thought to localise to the plasma membrane, including filopodial structures rather than cilia [10]. It has been suggested that CDON and BOC are distributed along cytonemes, which are actin-based filopodia in target cells [11].

Upon ligand binding, the SHH–PTCH1 complex moves from the primary cilia to the plasma membrane, undergoes endocytosis and is degraded in lysosomes. LDL-related protein 2 (LRP2) functions in the internalising and trafficking of various ligands after receptor binding, including the SHH–PTCH1 complex, and it is necessary for the patterning of the rostral ventral midline in mouse diencephalon [18].

The absence of PTCH1 in the ciliary membrane leads to activation of SMO, which forms a complex with kinesin-like protein KIF3a, after various modifications, and is translocated to the cilium. SMO can function outside the cilia but enrichment of SMO in the ciliary membrane appears necessary for its optimal function because a mouse mutant where SMO was not enriched in the cilia showed facial and skeletal defects similar (but less severe) to those in mice with complete loss of SMO function [19]. There is a negative feedback loop involving PTCH1, which can modulate HH signalling. HH activates PTCH1 and leads to de-repression of SMO, leading to an increase in PTCH1 and reduction in abundance of HH ligands [20]. The binding of PTCH1 by SHH releases the repression of SMO, leading to alterations of GLI transcription factors, GLI1–3, from full-length GLI to its repressor or activator forms. GLI 2 and 3 are bimodal, acting as activators or repressors in response to a HH ligand, but GLI1 only functions as an activator. The activator forms of GLI (GLI 1A) are seen with high levels of the SHH ligand, whereas repressor forms of GLI (GLIR) are seen in the absence of the SHH ligand [12]. This results in different gradients of repressor and activator forms of GLI 2/3 [21], which elicits different cell fates in target cells to establish cell patterning in organs. SHH can establish a concentration gradient when signalling over long ranges as shown in human forebrain organoids [22]. Repressor and activator forms have different functions in different organs [5]. GLIA upregulates HH target genes [23], while GLIR binds to promoters of HH target genes to block transcription [1]. Target genes differ for different cell types [10] so that the effects of SHH vary in different organs. Protein kinase A (PKA) and SUFU (Suppressor of Fused) repress GLI transcription factors when SMO is inactive, leading to proteolytic processing of GLI proteins into repressor forms [13,24]. SUFU can block nuclear translocation of GLI proteins [1,25] and is itself regulated by the SHH–PTCH1 complex. SUFU is also a negative regulator of the Wnt pathway [24]. 

## 3. Non-Canonical Sonic Hedgehog Signalling and Modifiers of Signalling

SHH can activate PTCH1 in two ways: via a protein–protein or a lipid–protein interface, the latter involving the palmitoylated n-terminal peptide of SHH and cholesterol modified C-terminus of SHH. SCUBE2 sequesters SHH lipid moieties and blocks the lipid-protein interaction of SHH with PTCH1 [6]. The amino terminus part of SHH lacking both palmitate and cholesterol moieties can bind to PTCH1 with high affinity and inactivate PTCH1 but with reduced potency for signal transduction. This may have different roles or functions at different concentrations of SHH [26]. The surface of the n-terminal moiety of SHH that interacts with PTCH1 via a protein–protein interface overlaps with the site that binds heparan sulfate proteoglycans, SHH co receptors CDON/BOC and GAS1 and HHIP [26].

Different forms of non-canonical SHH signalling are seen. These can be mediated by PTCH1 independent of SMO and GLI, which influence the cell cycle and can promote apoptosis [1,27]. GLI-independent SHH signalling can occur downstream of SMO, with modulation of Ca^2+^ and actin cytoskeleton [28]. SMO-independent GLI activation can also occur [29]. SMO and GLI can be activated by other signalling pathways [1,29]. SMO-independent, PTCH1-dependent non-canonical SHH signalling can stimulate other pathways such as Wnt [30]. Mouse models with reduced *Shh* show absent floorplate at E9.5, cyclopia, proboscis, microcephaly, heart, lung, kidney and foregut malformations, marked anomalies of facial bones, vertebral columns, ribs and absence of digits [7]. *Smo* mouse mutants only survive to E9.5 and show cyclopia, holoprosencephaly, linear heart tube and open midgut [31]. Mice lacking SHH function die before birth [7] but mutants lacking *Smo* die around E9.0 [32]. Loss of SMO abrogates all forms of HH signalling (SHH, IHH and DHH) and other functions of SMO and is more severe than loss of only SHH function. There is some level of redundancy between the HH proteins such as between SHH and IHH [31]. In humans, most first trimester losses are thought to be due to major genetic defects but genetic investigations are not usually conducted in such cases, except in recurrent pregnancy losses, so that the impact of major gene abnormalities (such as large defects of *SHH*) in human fetuses is difficult to document. Some forms of non-canonical SHH signalling do not require primary cilia [1,33], as the signalling proteins are located outside of the cilia. In non-canonical SHH signalling, SHH guides axons by binding BOC and possibly also PTCH1, turning the growth cone towards SHH in mouse spinal cord [34]. In the cerebral cortical layers, BOC is needed for SHH-dependent synapse formation [21]. SHH can initiate ciliogenesis and autophagy in various cell types, including primary human fibroblasts, primary cortical neurons, by a non-canonical pathway involving SMO [35].

In addition, SHH signalling can be affected by environmental factors such as cholesterol. In the absence of SHH, PTCH1 binds to cholesterol and pumps out cholesterol molecules, but when PTCH1 is degraded in the endosome, SMO is modified with cholesterol. Cholesterol modification of SMO is needed for its complete activity and localisation of the protein (reviewed by [10]). Cholesterol binding at the extracellular cysteine-rich domain is needed for full activation of SMO at the molecular level [10]. Cholesterol access in cell membranes can regulate components of SHH signalling [36]. DHCR7 (7-dehydrocholesterol reductase, the final enzyme in formation of cholesterol) can activate the SHH pathway [37].

Signalling pathways are also affected by other modulators, such as extracellular vesicles and miRNA [38]. For example, asymmetric accumulation of extracellular vesicles in mouse embryos can activate the non-canonical HH pathway [39] and different classes of extracellular vesicles have different accessory molecules that can modify the effect of SHH in target cells [8,40]. Glycosaminoglycans such as heparan sulfate proteoglycans can modulate SHH signalling [41] by influencing release, dispersal and reception of HH ligands [13], possibly as chaperones during diffusion of the SHH ligand [11], allowing formation of the morphogen gradient [42]. Differences in the extracellular matrix in different organs can modulate the effects of morphogens such as SHH. The extracellular matrix also changes during development, often from a more myxoid and cellular matrix to a more collagenous and less cellular matrix, presumably also affecting signalling pathways.

## 4. Sonic Hedgehog and Holoprosencephaly

Mutations in *SHH* were the first genetic causes to be found in human holoprosencephaly [43] and *SHH* mutations are one of the more common mutations in human patients with non-syndromic holoprosencephaly spectrum ([44,45,46]. Its genetic causes are summarised in Table 1 and its syndromes were reviewed by Kruszka and Muenke [45]: holoprosencephaly is most commonly seen in trisomy 13 and less often with other aneuploidies, trisomy 18, 21 and 22. It rarely occurs in triploidy. Holoprosencephaly occurs in 13q deletion syndrome (*ZIC 2*), 18p deletion syndrome (*TGIF1*), Smith Lemli Opitz (SLO, *DHCR7*), Hartsfield syndrome (*FGFR1*), Steinfeld (*CDON* or variants), Culler Jones (*GLI2*), hydrolethalus (*HYLS1* and *KIF7*) and Pallister-Hall (*GLI3*). *PTCH1* mutations are also associated with holoprosencephaly [47]. Most of the genes involved cluster around a few signalling pathways especially the SHH pathway, including SHH, its receptor PTCH1, TGIF (a regulator of SHH, [48]), SIX3 (regulator of SHH, [49]) and ZIC2 (needed for activation of NODAL (upstream of SHH) and ciliogenesis, [50]).

Holoprosencephaly involves early defects in midline structures of the developing brain [51]. In mouse models, SBE7, a SHH brain enhancer, directs SHH expression in the prechordal plate and ventral midline of the forebrain. Early SHH release from the prechordal plate activates SHH signalling in the overlying forebrain [52]. Mice lacking SBE7 showed a loss of *Shh* in the prechordal plate and ventral midline of the forebrain and developed defects resembling holoprosencephaly [52]. In human embryos, SHH is expressed ventrally in the notochord, floorplate of the spinal cord and hindbrain [4].

Holoprosencephaly is a complex malformation of the cerebrum characterised by incomplete separation of the hemispheres of varying severity (Table 2), and is often manifested in associated facial anomalies that reflect the underlying brain malformation, especially in *SHH* associated holoprosencephaly although not with *ZIC2*, which shows a characteristic pattern of facial anomaly regardless of the brain malformation [53]. This is possibly because facial development is also regulated by SHH [54] as well as other signalling pathways, (Wnt PDGF, Notch) which are also mediated in primary cilia [46] (reviewed by [55]). The genetic aberration is thought to occur between the 18th and 28th day of gestation (reviewed in [46,56]). In the most severe form, there is one cerebral lobe with fused deep gray nuclei, absent corpus callosum and absent olfactory bulbs and tracts (alobar holoprosencephaly). In the next most severe form, semilobar holoprosencephaly, the left and right frontal parietal lobes are fused and there is incomplete separation of the cerebral hemispheres and varying separation of the deep gray nuclei. The olfactory bulbs and tracts are absent or hypoplastic; the corpus callosum is absent; and there is varying non-separation of the deep gray nuclei. In lobar holoprosencephaly, the cerebral hemispheres are fully developed except that the frontal lobes are joined anteriorly in the midline, the corpus callosum can be absent, hypoplastic or normal and the deep gray nuclei are separated. In arhinencephaly, the olfactory bulbs are absent. In another variant called middle interhemispheric variant, the posterior frontal and parietal lobes fail to separate, and the corpus callosum is incompletely formed: it has a normal callosal genu and splenium but the callosal body is absent. There are also microforms of holoprosencephaly including facial midline anomalies with normal brain structure or microcephaly. Associated facial anomalies include cyclopia, proboscis, synophthalmia, hypotelorism in alobar holoprosencephly. Less severe forms (semilobar or lobar holoprosencephaly) may be associated with midline cleft lip and flat nose.

SHH is the main Hedgehog ligand in the brain [12] and has various roles during brain development, including proliferation of brain cells, axon guidance and positional identity of stem cells [21]. SHH is involved in late neuronal development in mice [52]. SHH, SMO, PTCH1 and GLI proteins are prominently expressed in second trimester human fetal cerebral cortex and SHH, SMO and GLI2 show similar expression patterns [57]. The distribution of *SHH* mRNA has been studied in the human fetal brain [58] and found in early (8–10 week gestation) human ventral forebrain, including midline structures such as the thalamus and hypothalamus and basal ganglia but less prevalent in dorsal areas. It is secreted by choroid plexus cells, meningeal vascular smooth muscle and endothelial cells. Proteins secreted by the choroid plexus into the cerebral ventricles can circulate in the cerebrospinal fluid and influence adjacent tissues such as progenitor cells in the ventricular zone. In weeks 15 to 17, the density and distribution of *SHH* transcripts increase significantly in the forebrain, and *SHH* expression is higher in rostral versus caudal brain structures. *SHH* transcripts are found in proliferating radial glial cells and glutaminergic neurons. In the developing cerebral cortex, neural progenitor cells from the ventricular zone migrate along radial glial fibres to the cortical plate, which later forms the cerebral cortex. *PTCH1*, *BOC*, *GAS1* and *CDON* are expressed in human radial glial cells in the cortical ventricular zone, but *CDON* and *GAS1* expression are higher in the cortical plate than *BOC*, suggesting that *CDON* and *GAS1* have additional roles in cortical development [58]. It appears that high concentrations of SHH induce cell fate specification and patterning while low levels regulate proliferation [58]. There are epigenetic gains in genes needed for human corticogenesis compared to mice or monkeys [59]. In humans, the loss of one *SHH* allele is sufficient to cause holoprosencephaly, whereas in *shh*^−/−^ mice the loss of both alleles is needed to produce a similar phenotype [43]. This is possibly because SHH expression is more widespread in the larger and more complex human cerebral cortex compared to the mouse cortex [58], meaning that SHH functions are relatively more impacted by mutations. Gene dosage also influences the phenotype in human patients. In a large study of human patients, truncated mutations involving the N-terminus of SHH were shown to be more likely to cause holoprosencephaly than other types of mutations [60]. However, more patients with *SHH* mutations did not show holoprosencephaly, compared to patients with holoprosencephaly [60]. It is known that the same mutation can lead to different phenotypes in the holoprosencephaly spectrum within the same family with markedly different severity [43,44,61,62], even in monozygotic twins with the same *ZIC2* mutation [44]. A possible explanation is that holoprosencephaly seems to reflect a multi-hit and multigenic pathology, requiring two or more events involving different genes and environmental factors [44,56,62]. In mice, the removal of one copy of *Shh* on its own does not cause malformations, but the loss can cause semilobar holoprosencephaly if one copy of *Six3* is also deleted [63]. In human patients, the loss of one copy of *SHH* or one copy of *SIX3* can cause holoprosnecephaly, but *SHH* and *SIX3* mutations do not seem to occur together [64]. 

Genetic studies have found that although the normal parents and a child with holoprosencephaly share the same mutation, for instance in *SHH*, the child has additional mutations in another gene such as *TGIF* [62]. As many holoprosencephaly genes have not yet been found, and as clinical studies usually search for known gene mutations, it is possible that the changes in *SHH*-associated holoprosencephaly are actually due to mutations in more than one gene. There is variation in gene expressivity and penetrance, which might depend on one’s genetic background. This is also seen in animal studies. For example, *Cdon*^−/−^ mouse mutants show a different holoprosencephaly phenotype and midfacial defects, depending on the inbred strains [55], and there is probably a similar explanation in human patients. Even in families with the same mutation, the phenotype might vary. It is noted that not all the cells and structures within an individual organ show the same changes in patients. It has been suggested that this may be due to variations in signalling such as during assembly and disassembly of cilia during different phases of the cell cycle so that adjacent cells in the same organ can receive different signalling patterns [65]. It has also been suggested that PTCH1 (functioning in cilia) might inhibit SMO enzymatically in an ultrasensitive regime to remove cholesterol from the extracellular cysteine-rich domain of SMO to deactivate SMO, such that PTCH1 mutants with holoprosencephaly might show a reduced affinity for the SHH ligand, an increased catalytic activity or an increased affinity for SMO [66]. Pooled data from animal and human studies can be analysed to detect trends and common factors and to search for explanations although animal genetic models might not precisely reflect the changes in human patients with similar genetic defects [10]. Additionally, there are structural differences and differences in expression of signalling proteins such as GLI and SHH in human and animal tissues, especially in the brain [17,21,58], which means that the developmental pathways are also probably different. The developing human brain has additional cells, such as outer radial glia, which are present to a limited extent in mice. Structural differences are also seen, for example: the inner fibre layer and outer subventricular zone in the developing human brain are not seen in the mouse brain. These structural differences allow for the expansion of neuronal output and brain size in humans. Mouse models with mutations in genes that cause human microcephaly do not show the same anomaly [67].

It has been proposed that the phenotypic variability in *Gas1* and *Cdon* mutant mice, which display a range of holoprosencephaly phenotypes, might be partially caused by the stochastic changes in establishment and response to the SHH morphogen gradient in a complex developmental program, where *Shh* is expressed in a temporal sequence as neural tissues develop with additional differences in the expression of multiple HH receptors also contributing to variability in holoprosencephaly severity [15]. Multiple mutations in HH co-receptors have been identified in human patients with holoprosencephaly [15], including in *GAS1* in combination with *SHH* mutation [60] and *CDON* which usually results in the microform type of holoprosencephaly [46].

In holoprosencephaly patients with *SHH* mutations, extra-neuroanatomical/related facial anomalies were rare, but cardiac and genitourinary anomalies were found in a small percentage of the patients, including those without frank holoprosencephaly [60]. An earlier study reported that limb, vertebral or gut anomalies were not usually seen in patients with mutations in *SHH*, unlike in the *Shh*^−/−^ mouse model [4]. The reasons for the mild and variable changes in the internal organs are possibly similar to those for the brain.

The blockade of SHH by cyclopamine (an antagonist that binds SMO) also causes holoprosencephaly [68], which is evidence that SHH signalling is also involved. Indian hedgehog (IHH) had redundant roles with SHH and was co-expressed (or expressed in adjacent cells) in the node and some endodermal tissues; compound *Ihh* and *Shh* mutants showed a similar phenotype to *Smo* mutants but a more severe phenotype than *Shh*^−/−^ or *Ihh*^−/−^ mutants [31].

The deletion of *Gas1* or *Cdon* results in holoprosencephaly phenotypes but *Boc* deletion in a *Gas1* null background partially rescues the holoprosencephaly and facial defects [15]. *Boc* deletion in mouse mutants with background genetic defects in the other co-receptors do not show holoprosencephaly. However, the combined loss of *Boc* and *Gas1* causes severe defects in the developing digits, which suggeste that Boc has tissue-specific functions. The deletion of *Gas1* in mice causes microform holoprosencephaly with partial loss of SHH signalling in target cells, but the additional loss of a single *Shh* allele in *Gas1*^−/−^ mice caused a more severe phenotype [69], showing that GAS1 also interacts with SHH.

### Environmental Factors and Holoprosencephaly

It has been suggested that environmental factors such as maternal hypocholesterolemia in pregnancy might increase the risk of holoprosencephaly in offspring [44,70]. Administration of cholesterol inhibitors in early gestation caused holoprosencephaly-like features in animal models [70]. Endogenous cholesterol can activate SMO and act synergistically with SHH [71], but lowered cholesterol can suppress SMO activation [16]. Patients with Smith–Lemli–Opitz (SLO) syndrome, who have mutations in dehydrocholesterol reductase 7 (*DHCR7*), have decreased available cholesterol. Rare cases (about 5%) of severe SLO partients exhibit holoprosencephaly-like features [46] without mutations in the main holoprosencephaly genes *SHH*, *ZIC2*, S*IX3* and *TGIF* [72]. 

Alcohol accentuated the severity of the holoprosencephaly phenotype in *Cdon*^−/−^ mice [55], and maternal diabetes and retinoic acid exposure are other environmental risk factors [51,63]. High ethanol exposure early in gestation can cause holoprosencephaly in mice [51] and is synergistic for holoprosencephaly in mice with *Cdon* mutations [73]. Exposure to alcohol at a critical time can cause holoprosencephaly in mice heterozygous for *Shh* and *Gli2*, while *Shh*^+/−^ and *Gli2*^+/−^ mice would otherwise have a normal phenotype [74]. A study based on patient questionnaires suggested that environmental factors such as maternal diet and exposure to chemicals can modify the severity of holoprosencephaly in genetically susceptible patients [75]. It is thought that poorly controlled maternal diabetes and excessive alcohol consumption during pregnancy can cause oxidative stress, which downregulates *SHH* expression and contributes to phenotypic variability in patients with *SHH* mutations [76]. Gene–gene interactions are rare compared to gene–environment interactions in human patients with holoprosencephaly [77]. It rarely occurs in fetuses of diabetic mothers with poor glycaemic control and there is a small increase in incidence of VACTERL association (a non-random association of malformations involving Vertebral, Anal, Cardiac, Tracheo-Oesophageal, Renal and Limb) type findings in offspring of diabetic mothers [78]. Mouse models with deficiencies in hedgehog or ciliary function show some features similar to human VACTERL association, but it is probably multifactorial and includes different entities [78]. A study of exome sequencing in a case of VACTERL revealed multiple genetic variants [79].

## 5. Human Ciliopathies

Primary cilia are found on most cell types. They are microtubule-based organelles that project from the cell surface and can be a platform for transduction of several signalling pathways. Complex mechanisms have evolved for the bidirectional transport of proteins within cilia, which contain various proteins for the anterograde and retrograde transport of signalling molecules (intraflagellar transport—IFT). Anterograde IFT affects cilia assembly, while retrograde IFT affects cilia length and function. IFT-B and kinesin 2 are the main proteins for anterograde transport while retrograde transport uses IFT-A and dynein. IFT are needed for genesis, maintenance and function of cilia [80]. There is a transition zone and a basal body in the cilia that function as a gate to allow proteins in and out of cilia. However, at least some of these proteins that normally regulate hedgehog signalling within primary cilia can also function outside the primary cilia. For example ARL13B (ADP ribosylation factor-like GTPase 13B), a GTPase needed for proper ciliary function [81] and mutated in some cases of Joubert syndrome, can also function cell-autonomously for axonal guidance in mouse spinal cord development, outside of primary cilia, in non-canonical SHH signalling [34]. A mouse model with ARL13B outside cilia shows short cilia without ARL13B, but normal SHH signalling without the developmental defects seen in *Arl13b*^−/−^ mouse models [82].

Defective IFT in mouse mutants can cause abnormal SMO localisation in cilia [83]. Dynein-2 is involved in retrograde IFT in cilia [16]. In mutants with defective retrograde cilia traffic, SMO accumulates within cilia, indicating that it usually exits via retrograde IFT [84,85]. Cilia are also required to process full-length GLI3 to GLI3R in the developing mouse cerebral cortex [86].

Primary cilia act as cell antennae that receive chemical and mechanical signals from the extracellular environment for transduction into the cells [10]. Primary cilia also regulate cell cycle, mediate several signalling pathways, cell differentiation, cell-cell communication and autophagy [83]. Disorders involving cilia are termed ciliopathies. Ciliopathies, however, show variable effect, probably due to associated modifiers [16].

Primary cilia assembly and disassembly are coupled to the cell cycle especially in cycling progenitor cells [12]. There are likely gaps in HH signalling at times when a cell lacks a cilium [65]. During cell division, the mitotic spindle is formed by centrioles, which also replicate during cell division to form a mother and a daughter centriole in each newly formed cell. After cell division, the mother centriole is transformed into a basal body for docking to the cell membrane and for the formation of the primary cilia (reviewed by [23]). IFT and other proteins are involved in ciliogenesis. 

Apart from SHH signalling, primary cilia facilitate other signalling pathways, including G-protein-coupled receptors (GPCRs), Wnt, PDGFRα, TGFβ/BMP, Notch, Hippo, mTOR, FGF, EGF, IGF pathways [12,87,88]; therefore, ciliary disorders can also interfere with other ones. For example, mutations in IFT80 can cause aberrations in both Wnt and HH signalling, resulting in abnormal chondrocyte differentiation [89]. Of particular interest, Wnt, BMP and SHH signalling appear to function together to regulate several aspects of the central nervous system [21,90]. During development, Wnt and BMP often form opposing concentration gradients to SHH where these molecules act as powerful morphogens that drive concentration-dependent cell differentiation. For example, opposing gradients of BMP and SHH appears to establish the dorsal–ventral axis within neural tube development [7,90,91]. SHH signalling directly interacts with other pathway signalling, for example crosstalk occurs between SHH and especially FGF, Wnt, TGFβ signalling [21,28]. Furthermore, both Wnt and BMP appear to repress *Shh* expression, while SHH appears to repress *Wnt* expression [92]. There are components that are present in more than one pathway, for example: the co-receptor CDON also interacts with proteins of the NODAL pathway, which is upstream of SHH in the patterning midline structures. Defects of NODAL also cause holoprosencephaly in mice [73]. LRP2 is a clearance receptor for internalising multiple ligands including sex hormone binding protein, and vitamin carriers such as vitamin D and retinol binding proteins and several signalling molecules such as SHH [93].

Mutations of ciliary genes are thought to underlie polydactyly, which is seen in some syndromes as GLI processing occurs in primary cilia (e.g., in Bardot–Biedl and Meckel–Gruber syndromes) [94]. SHH functions as a morphogen in this context, where concentration of SHH and the length of exposure to SHH appear to specify type and identity of digits [94,95]. The ratio of GLI3R to GLI3A appears to control the digit type and number [28]. SHH affects cell proliferation by controlling expression of genes that encode cell cycle regulators such as D cyclins [94]. In mice, Wnt7a and retinoic acid contribute to the regulation of SHH expression. Similar to holoprosencephaly, the same genetic mutation in the same family can show a different phenotype. In human patients, ciliary defects were seen in hepatic ductal plates showing ductal plate malformations, but not in normally developed bile ducts and ductal plates, and fibroblasts from one patient showed reduced GLI2 compared to controls [96]. However, abnormal differentiation of biliary epithelial progenitors preceded ductal plate malformation and ciliary defects [97]. For example, in Meckel syndrome, a more severe ciliopathy with more pronounced and more frequent malformations, there was variable loss of cilia and the evolution of ductal plate malformations in different parts of the same liver [97]. The effect of morphogens might trigger a threshold effect, above which an anomaly is usually seen and below which there is no malformation. Even in animal experiments, not all cells or structures responded in the same way to the same experimental stimulus. For example, the same experimental manipulations in chick embryos did not cause limb malformations in all embryos, although trends were noted [98].

## 6. Short-Rib Polydactyly Syndromes

SRP refers to a group of autosomal recessive skeletal dysplasias caused by disorders of primary cilia, with or without polydactyly. This group of disorders is characterized by shortened ribs, causing a constricted thorax. A “trident” appearance of the acetabular roof is a radiological sign often used to support this diagnosis. This group includes SRP type 1/3 (Saldino–Noonan/Verma–Naumoff), SRP type 2, SRP type 4, SRP type 5, SRP unclassified and asphyxiating thoracic dysplasia/Jeune syndrome [99]. This group used to include Ellis van Creveld syndrome, with the alternative designation “short rib thoracic dysplasia,” which is still in clinical use, but Ellis van Creveld is not considered an SRP. Non-skeletal malformations include cleft lip/palate, and anomalies of the brain, eye, heart, kidneys, liver, pancreas, intestines and genitalia. Some of these anomalies are difficult to diagnose in human fetuses in early gestations when terminations might be performed because the changes are usually mild, poorly developed and difficult to differentiate from normal variations or non-specific changes. For example, a slightly short femur can be seen in growth restriction, a few renal tubules in the second trimester might be mildly dilated in fetuses without known genetic abnormalities, and mild anomalies of hepatic ductal plates can be difficult to diagnose without special studies to assess signalling pathway proteins. The assessment of mid-face hypoplasia can be subjective especially in mid gestation or younger fetuses as biometric measurements are lacking for some features.

Genetic mutations in several genes controlling ciliary proteins have been found in the SRP [99], including those involving IFT (such as IFT80, [100]; IFT52, [101]), dynein motor (such as DYNC2H1), centrosomal proteins such as KIAA0586, WDR (such as WDR35, [102]). Abnormalities in ciliary proteins can affect cilia structure and function and have been linked to various defects in SHH signalling [103]. These abnormalities have been found in various human genetic syndromes [103]. Mouse mutants with abnormalities in ciliary proteins also show similar defects [104]. For example, mice with defects in anterograde IFT proteins such as KIF3a, IFT172 and IFT88, or in proteins of the basal body or transition zone show spinal cord defects similar to that of embryos lacking *Gli2* and *Gli3*. However, in other mutants with abnormal cilia, SHH signal transduction can be partially over-activated. Mutations involving retrograde IFT genes (such as *Dync2h1*) cause variable defects in mouse mutants, even if structural anomalies are present. These data show the complex relationship between SHH signalling and individual cilia proteins [12] SHH signalling is also involved in the patterning of the limbs in combination with other pathways. Aspects of limb patterning, including formation of the fingers and forelimbs, appear to be dependent on primary cilia, involving SHH and other pathways including IHH [105]. SHH expressed in the zone of polarising activity interacts with the overlying apical ectodermal ridge. Patients with SRP infrequently show mild brain malformations within the holoprosencephaly spectrum [12] although canonical vertebrate SHH signalling is mediated in the primary cilium. 

The most common neurological problems seen in human patients with ciliopathies include intellectual disability, epilepsy, corpus callosum anomalies and ataxia. Outward signs include abnormal brain size (microcephaly or macrocephaly), hydrocephalus and hypothalamic hamartomas [17]. In animal models with cilia defects, the main abnormalities were neural tube defects (including exencephaly, anencephaly, spina bifida, encephalocoele), corpus callosum agenesis or dysgenesis, hydrocephalus, cerebellar hypoplasia (including vermal agenesis and brainstem anomalies as in Joubert syndrome) and probably some forms of microcephaly [106]. Brain anomalies in SRP syndromes are relatively uncommon. Arhinencephaly (absence of olfactory bulbs) and agenesis of the corpus callosum have been described in SRP patients with *KIAA0586* mutations [12]. In animal models, the knockdown of *Ift80* and *Kif7* (genes that can cause Jeune syndrome and SRP3) can cause impaired ciliary transduction without loss of cilia, resulting in reduction in radial progenitor cells and delayed neuronal migration [107]. However, morphological studies of mouse cortical neurons suggested that immature cilia (lacking axonemes, the microtubular backbones) only form in the neuronal cells after neuronal migration is complete and that ciliary maturation mostly occurs post-natally [108]. This may explain the milder brain phenotype caused by IFT mutations found in many cases of SRP, while mutations involving the ciliary basal body and gate have a larger impact on brain malformations.

Human ciliopathies show a different spectrum of brain and other anomalies. Holoprosencephaly is unusual and mild or not seen although mid-face abnormalities can be present. This suggests that other factors apart from the canonical HH pathway are likely to be involved in holoprosencephaly malformation associated with *SHH* mutations. *GLI*2 mutations are mostly associated with mild midfacial abnormalities, hypopituitarism and polydactyly with genito urinary tract anomalies in some cases rather than patent holoprosencephaly, despite abnormal HH signalling [109], with holoprosencephaly reported in only a minority (about 2%) of patients [110], possibly due to deletion of genes adjacent to *GLI2* [111]. This is unlike patients with *SHH* mutations who present with varying abnormalities in the holoprosencephaly spectrum. Loss of *Gli2* in a mouse model caused disrupted cell architecture in the midline and failure of closure of the neural tube. Cells in the midbrain of *Gli2* mutants had different shapes when compared to those from wild type mice. Similar findings were seen with induced activation of *Shh* through the midbrain and suggested that the defects were due to dysregulated *Shh* signalling [112]. In contrast, the complete absence of SMO or SHH in mouse models resulted in holoprosencephaly because SHH is required for cell migration and axonal guidance in the developing brain. Bi-allelic *SMO* variations causing the loss of SMO function in humans resulted in variable phenotypes, including hypothalamic hamartomas and microcephaly, shortening of limb bones, narrow chest and heart defects, probably due to defects in HH signalling, which was confirmed in studies of patient fibroblasts [113].

These data suggest that SHH mediation in holoprosencephaly can either be altered by cofactors such as BOC, which alters the severity of HH-mediated holoprosencephaly, [114], or by *SHH* mutations disrupting other parts of SHH signalling apart from canonical SHH signalling.

There are few syndromes where holoprosencephaly is associated with polydactyly, suggesting that these developmental processes are probably mediated by different mechanisms. An example is pseudo trisomy 13, where HH signalling wsa not shown to be involved in limited studies. Unfortunately, this is a rare syndrome and case reports have limited information. For example, Cordero et al. [115] reported a case of an infant with holoprosencephaly and polydactyly, which they investigated with a panel of FISH probes and a CGH array. They found that the usual mutations associated with human holoprosencephaly (*SHH*, *TGIF*, *ZIC2* and *SIX3*) and *GLI 3* were not present, and cholesterol studies were normal. Moreover, a report on overlapping holoprosencephaly–polydactyly syndrome and asphyxiating thoracic dystrophy lacked autopsy and genetic findings [116].

## 7. Comment

Data from animal and human studies suggested that cilia dependent canonical SHH signalling controls development of midline facial and brain structures. However, this signalling pathway is subject to modifying factors, such as co-receptors, cholesterol levels, maternal health and drug status, so brain abnormalities might not be seen in some fetuses with *SHH* mutations or abnormalities in primary cilia although mid-face anomalies and other malformations suggestive of SHH signalling aberrations were still noted. This is especially the case as *SHH* was not completely abrogated in human fetuses unlike in the experimental models. It appeared that the significant loss of SHH function was required for brain anomalies to develop. Postaxial polydactyly is often noted in SRP and finger abnormalities are found in association with ciliopathies and *DHCR7* mutations, suggesting that cholesterol processing was needed for the correct patterning of fingers. SHH signalling differed in different organs and the effect of its co receptors, such as BOC, was different in limb malformations and brain anomalies. Interactions with other pathways also affected the outcome. It is likely that holoprosencephaly was initiated by SHH aberrations causing midline facial and brain maldevelopment, but fully developed holoprosencephaly is affected by other factors, including non-canonical HH signalling outside the primary cilia, while polydactyly and limb anomalies appear to be more dependent on primary cilia. Animal and human studies complemented each other, but could not address some aspects of human development that differed from that in animal models. Brain organoids have been developed that are useful for investigating human cerebral development and disease [117]. Culture systems that more closely resemble the physiological environment in living organisms (microphysiological models) can also contribute [77].

In theory, it may be possible to prevent some abnormalities resulting from defective SHH signalling by activating the SHH signalling pathway in utero, which, in a mouse model, appeared to prevent congenital defects caused by defective SHH signalling [118]. However, this would be currently clinically impossible in humans. Firstly, the defect(s) in SHH signalling would need to be identified prior to specific developmental timepoints. Currently, these conditions are identified following development by ultrasonography or at birth. It might be possible to identify *SHH* and pathway mutations with parental genetic sequencing, but the same mutation can cause different phenotypes and this method would miss de novo mutations. In this context, avoiding SHH signalling mutations with pre-implantation genetic testing could be possible with in vitro fertilisation. 

Secondly, there currently are no safe or effective methods to activate SHH signalling in utero. An effective agonist for SMO (SAG) exists [68,118]; however, this compound would be unlikely to undergo human trials for rare developmental conditions. Furthermore, some *Shh* mutations in mice appear insensitive to SAG activation [19]. Genetic engineering techniques to correct *SHH* mutation(s) are also theoretically possible, however in utero genetic modification does not appear possible with current viral vector technology. Thirdly, activation of SHH signalling, which is required at specific spatial and temporal points during the developmental, and widescale non-specific activation of SHH signalling are likely to cause secondary developmental problems. Supporting this, the SMO agonist SAG appears to induce preaxial polydactyly in healthy mice following early gestational exposure [119].

Regarding SHH signalling, current drug development efforts are focused on inhibiting aberrant SHH/HH pathway activation in cancer. The small molecule inhibitors vismodegib and sonidegib are FDA/EMA approved SMO antagonists used to treat advanced basal cell carcinoma and other cancers [120]. Like cyclopamine, these compounds are teratogenic in humans and animals [121]. Several other SHH/HH signalling inhibitors were approved or were under experimental investigation as cancer treatments including the GLI inhibitors arsenic trioxide and GANT-61 [122], the Shh palmitoylation inhibitor RU-SKI 43 [123], and the monoclonal antibodies that block SHH binding/function [124].

It is apparent that there are many modifiers for genetic syndromes, including environmental factors, epigenetics and other genetic mutations, especially in complex signalling pathways where there are multiple interactions with other pathways. Investigation of the interactions and mechanisms of these modifiers would help clarify the variable inheritance observed in families with genetic mutations. This information can also help limit the severity and incidence of birth defects. Data from clinical studies and experimental research have already contributed to the amelioration of some birth defects. For example advising mothers about the dangers of high alcohol intake in pregnancy reduced the frequency of fetal alcohol syndrome. The data on interactions among different genes and genes and the environment are also helpful in prenatal counselling.

## Figures and Tables

**Table 1:** Genetic Causes of Holoprosencephaly.

Syndromes	Genetic Abnormality	Clinical Features
Chromosomal	Trisomy 13 (almost 50% of all cases holoprosencephaly)	Holoprosencephaly in 8–39%, cleft lip and palate, rocker bottom feet, postaxial polydactyly
	Trisomy 18, 21, 22	Holoprosencephaly infrequent
	triploidy	Holoprosencephaly rare, syndactyly, lethal in 1st or 2nd trimester
13q deletion	*ZIC2*	Holoprosencephaly, kidney, heart, eye, facial and limb anomalies
18p deletion	*TGIF1*	Holoprosencephaly rare
Smith Lemli Opitz	*DHCR7*	Holoprosencephaly about 5%
Hartsfield	*FGFR1*	Holoprosencephaly occasional, ectrodactyly
Kallman syndrome 2	*FGFR1*	Cleft lip and palate, hypogonadism and holoprosencephaly occasional
Steinfeld	*CDON*	Microform holoprosencephaly
Culler Jones	*GLI2*	Hypopituitarism, post axial polydactyly, holoprosencephaly unusual
Non-syndromic causes of Holoprosencephaly	Estimated incidence	
*SHH*	5–6%	
*ZIC2*	5%	
*SIX3*	3%	
*TGIF1*	<1%	
Less common		
*CNOT1*	<1.5%	
*DISP1*	<1.2%	
*FGF8*	<2.2%	
*FGFR1*	1.2%	
*DLL1*	<1%	
*PPP1R12A*, *RAD21*, *SMC1A*, *SMC3*, *STAG2*, *STIL*, *PTCH1*, *CDON*	Rare causes of holoprosencephaly	

**Table 2:** Clinical Forms of Holoprosencephaly.

Forms of Holoprosencephaly (in Decreasing Order of Severity)	Clinical Features
Alobar holoprosencephaly (most severe)	one cerebral lobe with fused deep gray nuclei, absent corpus callosum absent olfactory bulbs and tracts cyclopia, proboscis, synophthalmia, hypotelorism
Semilobar holoprosencephaly	fused left and right frontal parietal lobes, incomplete separation of cerebral hemispheres, varying separation of the deep gray nuclei. Absent or hypoplastic olfactory bulbs and tracts, absent corpus callosum, varying non separation of deep gray nuclei. midline cleft lip and flat nose.
Lobar holoprosencephaly	Fully developed cerebral hemispheres but frontal lobes are joined anteriorly in the midline, normal or hypoplastic corpus callosum separated deep gray nuclei midline cleft lip and flat nose.
Middle interhemispheric variant	failure of separation of posterior frontal and parietal lobes incomplete formation of the corpus callosum, with normal callosal genu and splenium but absence of the callosal body.
Agenesis of corpus callosum	Part or all of the corpus callosum can be absent
Microform holoprosencephaly	facial midline anomalies with normal brain structure or microcephaly.
Arhinencephaly	absent olfactory bulbs

## Abbreviations

ARL13B	ADP ribosylation factor like GRPase 13B
BMP	bone morphogenetic protein
BOC	brother of CDON
CDON	cell adhesion molecule-related/down regulated by oncogenes
COS2	COSTAL 2
DHCR7	7 Dehydrocholesterol reductase
DHH	Desert hedgehog
DISP1	Dispatched 1
DYNC2H1	Dynein cytoplasmic 2 heavy chain 1
EGF	Epidermal growth factor
FGF	Fibroblast growth factor
FGFR	fibroblast growth factor receptor
GAS1	Growth arrest specific 1
GLI	Glioma-associated (family of transcription factors)
GPCR	G protein coupled receptor
GTPase	Guanosine triphosphatase
HH	Hedgehog
HHAT	Hedgehog acyl transferase
HHIP	Hedgehog interacting protein
IFT	Intraflagellar transport
IGF	Insulin-like growth factor
IHH	Indian hedgehog
KIF	Kinesin family
LRP2	low density lipotein (LDL) receptor-related protein 2 (also known as megalin)
mTOR	mammalian target of rapamycin
PDGFα	Platelet derived growth factor alpha
PKA	Protein kinase A
PTCH	Patched
SBE7	Sonic Hedgehog brain enhancer 7
SCUBE2	signal peptide, CUB domain and EGF domain containing protein 2
SHH	Sonic Hedgehog
SIX3	SIX homeobox 3
SLO	Smith-Lemli-Opitz
SMO	Smoothened
SRP	Short rib polydactyly
SRTD	Short rib thoracic dysplasia
SUFU	Suppressor of Fused
TGFβ	Transforming growth factor beta
TGIF	thymine guanine-interacting factor
VACTERL	Vertebral Anal Cardiac Tracheo Oesphageal Renal Limb
WDR	WD40-repeat (a series of loosely conserved structural motifs comprised of about 40 amino acids, often terminating in tryptophan (W) and aspartic acid (D))
WNT	Wingless and Int-1
ZIC2	ZIC gene family 2
ZPA	Zone of polarising activity
